# High-Flow Oxygen through Nasal Cannula vs. Non-Invasive Ventilation in Hypercapnic Respiratory Failure: A Randomized Clinical Trial

**DOI:** 10.3390/ijerph17165994

**Published:** 2020-08-18

**Authors:** Yiannis Papachatzakis, Pantelis Theodoros Nikolaidis, Sofoklis Kontogiannis, Georgia Trakada

**Affiliations:** 1 Department of Clinical Therapeutics, School of Medicine, National and Kapodistrian University of Athens, Alexandra Hospital, 11527 Athens, Greece; yiannispapachatzakis@gmail.com (Y.P.); agupiela@gmail.com (S.K.); gtrakada@med.uoa.gr (G.T.); 2 Department of Occupational Therapy, School of Health and Caring Sciences, University of West Attica, 12243 Athens, Greece

**Keywords:** high-flow oxygen through nasal cannula, hypercapnicrespiratory failure, non-invasive ventilation, partial carbon dioxide arterial pressure

## Abstract

High-flow oxygen through nasal cannula (HFNC) provides adequate oxygenation and can be an alternative to noninvasive ventilation (NIV) for patients with hypoxemic respiratory failure. The aim of the present study was to assess the efficacy of HFNC versus NIV in hypercapnic respiratory failure. Patients (n = 40) who were admitted to the Emergency Department of Alexandra Hospital due to hypercapnic respiratory failure (PaCO_2_ ≥ 45 mmHg) were randomized assigned into two groups, i.e., an intervention group (use of HFNC, n = 20) and a control group (use of NIV, n = 20). During their hospitalization in the Intensive Care Unit, vital signs (respiratory and heart rate, arterial blood pressure) and arterial blood gases (ABG) were closely monitored on admission, after 24 h and at discharge. No difference between the two groups regarding the duration of hospitalization and the use of HFNC or NIV was observed (*p* > 0.05). On admission, the two groups did not differ in terms of gender, age, body mass index, APACHE score, predicted death rate, heart rate, arterial blood pressure and arterial blood gases (*p* > 0.05). Respiratory rate in the HFNC group was lower than in the NIV group (*p* = 0.023). At discharge, partial carbon dioxide arterial pressure (PaCO_2_) in the HFNC group was lower than in the NIV group (50.8 ± 9.4 mmHg versus 59.6 ± 13.9 mmHg, *p* = 0.024). The lowerPaCO_2_ in the HFNC group than in the NIV group indicated that HFNC was superior to NIV in the management of hypercapnic respiratory failure.

## 1. Introduction

Heated and humidified high-flow oxygen through nasal cannula (HFNC) has been developed over the past 2 decades, as an alternative to standard oxygen delivery systems [1,2]. The device delivers a flow rate up to 8 L/min in infants and 60 L/min in adults and allows adjustment of inspired oxygen independently of the flow rate. Traditional oxygen delivery systems—nasal cannula, non-rebreathing masks, masks with reservoir bags, and Venturi masks—allow varying levels of inspired oxygen reliant on the patient’s breathing pattern, peak inspiratory flow rate, delivery system, and mask characteristics [3,4]. Additionally, their inability to heat and humidify gas limits patient’s comfort and tolerability at high-flow rates.

Potential mechanisms of clinical benefit during HFNC use include enhanced and more reliable oxygen delivery, improvement of alveolar ventilation, decreased work of breathing, and facilitation of secretion removal, comfort and tolerability [5]. In infants, children, and preterm neonates, HFNC has become a first line treatment in several clinical situations of respiratory distress [6]. In adults, it has been used to treat hypoxemic respiratory failure, cardiogenic pulmonary edema, postoperatively and postextubation in do-not-intubate patients or during bronchoscopy [5,7].

To date, the literature supports the possibility to use HFNC as alternative to non-invasive ventilation (NIV) in some settings, while in others might be even superior [8]. It is also an alternative to standard oxygen as first line therapy in management of patients with acute respiratory failure [8]. NIV is strongly recommended in patients with acute-on-chronic respiratory failure associated with acute respiratory acidosis, the vast majority of whom meet the criteria for Chronic Obstructive Pulmonary Disease (COPD) exacerbation [9].

Recently, Lee MK et al. [10] revealed no difference of the 30-day mortality and intubation rate between NIV and HFNC, in severe acute exacerbation of COPD with moderate, hypercapnic, acute respiratory failure. In a retrospective study, Kim et al. [11] suggested that HFNC oxygen therapy was beneficial, even in respiratory failure Type 2, resulting in significant improvement of both oxygenation and hypercapnia. Bräunlich et al. [12] also indicated that HFNO leads to a flow-dependent reduction in PaCO_2_ in patients with stable hypercapnic COPD, due to a washout of the respiratory tract and a functional reduction in dead space.

Although cumulative evidence supports that HFNC is effective in patients with hypercapnia, randomized studies to compare HFNC vs. NIV in patients with acute, hypercapnic respiratory failure are missing. We conducted this prospective, randomized, controlled trial, involving patients admitted to the Emergency Department (ED), to compare the efficacy of HFNC versus NIV in the management of acute hypercapnic respiratory failure.

## 2. Materials and Methods

### 2.1. Population and Study Design

In our study, we enrolled patients who were admitted to the ED of “Alexandra” tertiary hospital, in Athens, Greece, because of acute respiratory failure type 2 (PaCO_2_ > 45 mmHg), from 1 January 2017 to 30 December 2018. The study protocol was approved by the Ethical Committee of our hospital, before the initiation of the study (Approval number: 41/21.06.2016). We obtained written informed consents from all involved participants (subjects who were able to consent or family member or other surrogate available) before enrollment.

Immediately upon arrival, forty patients (female, n = 21; male = 19; age, 77.0 ± 11.0 years) were randomized 1:1 in the intervention group A (use of HFNC, n = 20) and in the control group B (use of NIV, n = 20) and then hospitalized in the High Dependency Unit. The number of included patients was determined according to sample size calculation with regards to PaCO_2_ as outcome measure and considering the sample sizes of relevant studies (e.g., n = 33, [11]; n = 54, [12]). Underlying comorbidities were established in detail by using clinical history supported where appropriate with a review of available medical records. We excluded patients with known cancer or neuromuscular diseases or pH < 7.20. Children were excluded, too, since our ED is a health unit only for adults.

In all patients, we measured and recorded vital signs, arterial blood gases (ABGs), and comfort on admission to the unit, during the hospitalization period and at discharge. We also calculated APACHE score to determine every patient’s mortality risk. Predicted mortality risk provides an estimate of the risk of death without having to specify a primary diagnosis. Appropriate therapy using additional forms of respiratory support such as bronchodilators, diuretics, and antibiotics in each patient started upon admission. Endpoints to evaluate after HFNC or NIV therapy were intubation and mortality rate, length of hospitalization, duration of therapy, and possible differences between vital signs, ABGs, and comfort. During hospitalization, no patient needed transoral or transnasal intubation or tracheotomy.

### 2.2. HFNC and NIV Application

HFNC was delivered using an Optiflow nasal interface connected to the PT101AZ (Airvo2) humidifier (Fisher & Paykel Healthcare, Auckland, New Zealand). Therapy typically was initiated at a flow of 35 L/min, titrating flow upward if tolerated to 45–50 L/min, in order to maintain SaO_2_ > 90% or according to specific clinical orders. In addition, all patients from the HFNC group answered a questionnaire about comfort and tolerability during the first 12 h of their admission to the ED.

NIV was initiated by bi-level positive airway pressure (BiPAP) in the spontaneous/timed mode (Synchrony, Respironics, Inc, Murrysville, Pennsylvania) supplied with an identical set of masks. Expiratory and inspiratory pressures were gradually increased to the maximum tolerated over 1 h, in order to maintain SaO_2_ > 90%, or according to specific clinical orders.

### 2.3. Statistical Analysis

Qualitative variables are presented as absolute (n) or as percentage (%) frequencies and quantitative variables are presented as mean (SD) or as median (IQR). Kolmogorov–Smirnov was used to check normal distribution. Chi-square test or Fisher’s exact test was used for determining the relationship between two qualitative variables. Student’s *t*-test was used for determining the relationship between a quantitative variable and a bivariate variable for normal distribution and Mann–Whitney test was used when the quantitative variable did not follow normal distribution. Multivariate linear regression and backward stepwise linear regression were used for bivariate analysis in case that more than two variables had statistically significant difference. Regarding multiple logistic regression, odds ratio, 95% CI, and *p* values were presented. Analysis of variance for repeated measures was used for detecting differences in arterial blood gases. Mauchly test for sphericity was used if there was symmetry, and in case of asymmetry Greenhouse–Geisser was applied. The statistical significance level was set equal or less to 0.05, and the analysis was performed with IBM SPSS 20.0 (Statistical Package for Social Sciences).

## 3. Results

Forty patients were included in the study. All of them were admitted to our hospital because of Hypercapnic Respiratory Failure (PaCO_2_ > 45 mmHg). Twenty patients were randomized (1:1) in the intervention group (use of HFNC with warm humidified air) and 20 patients in the control group (use of NIV). There was no statistically significant difference between the two groups regarding age, gender, Body Mass Index (BMI), comorbidities, APACHE score, and predicted mortality risk (Table 1). In the whole population, 42.5% of the patients had chronic heart failure (CHF) and 62.5% had Chronic Obstructive Pulmonary Disease (COPD) while 42.5% had Diabetes Mellitus (DM). Although more patients in the intervention group had COPD in comparison with the control group, there was no statistically significant difference (*p* = 0.327).

In the Emergency Department, the patients had a mean Respiratory Rate (RR) of 23.9 ± 7.6 breaths per minute (A: 21.3 ± 8.7 vs. B: 26.6 ± 5.3, *p* = 0.023) and a mean Heart Rate (HR) of 87.5 ± 23.7 beats per minute (A: 87.1 ± 29.3 vs. B: 88 ± 17.1, *p* = 0.911). All were hypercapnic (mean PaCO_2_ 61.2 ± 10 mmHg, A: 60.4 ± 9.9 vs. B: 62.1 ± 10.3, *p* = 0.586). Arterial blood gases values upon admission and 24 h after hospitalization are presented in Table 2. Half of the patients (20 pts—50%) were already in Long Term Oxygen Therapy (LTOT) and 6 pts (15%) under NIV, at home. Patients under NIV in their residencies were represented more in the control group (*p* = 0.010) than in the intervention group.

The length of hospitalization did not differ in a statistically significant way between the two groups (mean 11.5 ± 8.5 days, A: 11.5 ± 7.8 vs. B: 11 ± 10.5, *p* = 0.655). Longer hospital stay was associated with male gender (*p* = 0.013), independently of the therapeutic procedure. Moreover, in-hospital severe complications—like acute renal or hepatic failure—and mortality rate did not differ in a statistically significant way between the two groups (Table 3). The median continuous—for 24 h—use of either HFNC or NIV was 2 ± 2 days (A: 2 ± 1 vs. B: 2 ± 9, *p* = 0.078). Three (3) patients (7.5%) from the control group changed from NIV to HFNC due to discomfort, nasal ulcer, and lack of cooperation. The answers of the HFNC group with regards to comfort and tolerability are presented in Table 4.

**Figure 1:**
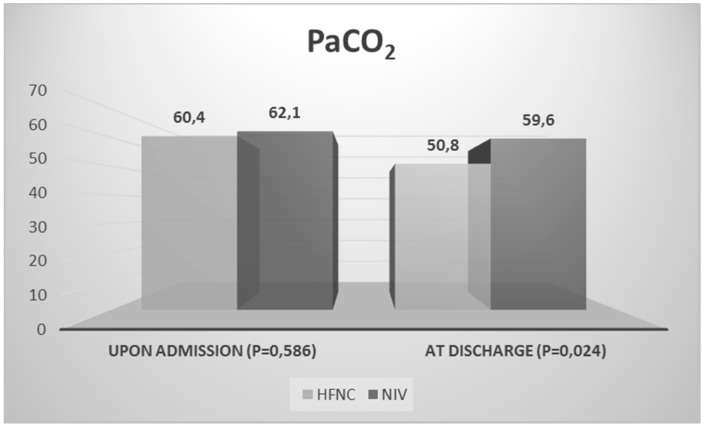
PaCO_2_ of the two groups upon admission to the hospital and after discharge.

At discharge from hospital, RR decrease was statistically significant in both groups (A: from 21.3 ± 8.7 to 15.7 ± 3.5, mean difference = −5.6, 95% CI, −8.3, −2.9, *p* = 0.0111 vs. B: from 26.6 ± 5.3 to 17.3 ± 4.6, mean difference = −9.3, 95% CI, −7.8, −3.4, *p* = 0.0001, breaths per minute). Instead, HR decrease was statistically significant only in NIV group (B: 88.0 ± 17.1 vs. 74.5 ± 11.4, mean difference = −13.5, 95% CI, −19.8, −7.3, *p* = 0.0452, beats per minute). Statistically, the mean value of PaCO_2_ in the intervention group was significantly lower in comparison with the control group (A: 50.8 ± 9.4 mmHg vs. B: 59.6 ± 13.9, mean difference = −8.8, 95% CI, −13.9, −3.7, *p* = 0.024). Although PaCO_2_ on admission and at discharge decreased more in the HFNC group than in the NIV group, the difference was not statistically significant (A: from 60.4 ± 9.9 to 50.8 ± 9.4 mmHg vs. B: from 62.1 ± 10.3 to 59.6 ± 13,9 mmHg, *p* = 0.080) (Figure 1). According to the analysis of variance for repeated measures, no statistically significant difference was observed between PaCO_2_ and age (*p* = 0.080), gender (*p* = 0.434), BMI (*p* = 0.454), prior NIV in their residencies (*p* = 0.220), days of hospitalization (*p* = 0.960), duration of either NIV or HFNC use (*p* = 0.991), change from NIV to HFNC (*p* = 0.608), or in which group patients were initially randomized (*p* = 0.080).

## 4. Discussion

In our study, HFNC oxygen therapy led to a significant decrease of PaCO_2_ levels and showed similar in-hospital severe complication and mortality compared with NIV, in patients with respiratory failure type 2. To our knowledge, this is the first randomized, controlled clinical trial assessing HFNC versus NIV, in patients with acute hypercarbia in an emergency department. According to current guidelines, NIV is the treatment of choice in patients with hypercapnic respiratory acidosis, whereas HFNC oxygen therapy is indicated in patients with hypoxemic respiratory failure.

Díaz-Lobato et al. [13] first reported on a 65-year-old woman suffering from amyotrophic lateral sclerosis (ALS), with acute hypercapnic respiratory failure, who was successfully treated with HFNC. Then, Millar et al. [14] presented a 57-year-old woman suffering from COPD, with hypercapnic respiratory acidosis and unable to tolerate conventional NIV, who was also successfully treated with HFNC. These two cases initially demonstrated clinical benefits with HFNC therapy in hypercapnic patients because of reduction in PaCO_2_.

Further to these preliminary data, Bräunlich et al. [12] assessed that different flow rates of HFNC in patients with stable, hypercapnic COPD, resulted in a flow-dependent reduction in both PaCO_2_ and rapid shallow breathing index [12]. In another study, HFNC in patients with stable, oxygen-dependent COPD also led to a significant reduction in transcutaneous (Tc) CO_2_ levels and RR, with corresponding increases in tidal volume (Vt), end-expiratory lung volume (EELV), and inspiratory/expiratory (I:E) ratio, without changes to minute volume (MV) [15]. Moreover, HFNC therapy in severe acute exacerbation of chronic obstructive pulmonary disease (AECOPD), with moderate hypercapnic, acute respiratory failure, demonstrated a significant decrease in PaCO_2_ in addition to pH and PaO_2_ improvements, after 6 and 24 h of application, with similar endpoints including the intubation rate and 30-day mortality compared with NIV group [10].

Our data were in accordance with those previously published. In our study, although both therapies, HFNC and NIV, improved hypercapnia, at discharge from hospital, PaCO_2_ in the HFNC group was, statistically, significantly lower in comparison with the NIV group. The observed reduction of RR probably enhanced the effectiveness of breathing and consequently improved ventilation and perfusion matching. Moreover, HFNC was comfortable and well tolerated, as none of the patients in this group changed from HFNC to NIV. On the contrary, 3 patients from the control group changed from NIV to HFNC due to discomfort, nasal ulcer, and lack of cooperation. Finally, severe complications—like renal or hepatic failure—and mortality rate during hospitalization were similar among the two groups.

The potential mechanisms of clinical benefit during HFNC use were recently reviewed [6]. HFNC is associated with greater overall comfort and tolerance, lower dyspnea scores, and reduced mouth dryness [16]. Breathing warming and humidified inspired gas helps to maintain adequate mucosal function and preserve the rheology and volume of secretions, facilitating expectoration and preventing atelectasis [17]. Additionally, breathing warming and humidified gas may help to avoid the bronchoconstriction effect of cold, dry gas, reducing the metabolic cost of the work of breathing [18]. Due to high inspiratory flow rates that do not permit entrainment of room air during patient inspiration, HFNO assures more reliable delivery of desired FIO_2_ [19]. Moreover, the continuously flushed-out CO_2_ from the nasopharynx eliminates dead space, further improving the ventilatory efficiency [20]. Combined with the generation of a small positive end-expiratory pressure (PEEP), which may counterbalance auto-PEEP, the work of breathing is further reduced and allows an improved ventilation and perfusion matching [21]. In healthy volunteers and in postcardiac surgery patients, HFNC reproduces the favorable effects of Continuous Positive Airways Pressure (CPAP) by increasing tidal volume and reducing the respiratory rate, with steady minute volume [22,23].

Our study has some limitations. First, we performed a single center study and not a multicenter one. Second, the number of enrolled patients was small. Third, we included all patients with respiratory failure type II, independently of the underlying disease. It should be highlighted that the relatively short duration (~two days) of respiratory support by NIV and HFNC considering the average length of stay was attributed to an amelioration of the condition of the patients leading them to conventional oxygen therapy. However, the main advantage of our study was the immediate—upon arrival in the emergency department (ED)—randomization 1:1 in HFNC or NIV therapy. Thus, initiating therapy in ED should not influence clinical outcomes preventing the inclusion of patients likely to improve with other respiratory interventions.

## 5. Conclusions

According to our data, we suggest that HFNC could be an alternative treatment of hypercapnic respiratory failure, especially when NIV is not well tolerated. Further multicenter and randomized controlled studies are needed to determine the optimal use of HFNC in respiratory failure type II in relation to NIV.

## Figures and Tables

**Table 1:** Demographics, Apache score, predicted death rate, and comorbidities in the study population.

	Total Population	Patients		*p* Value
		Intervention Group (n = 20)	Control Group (n = 20)	
**Age (years, SD)**	77.0 (11.0)	76.0 (13.4)	78.1 (8.1)	0.544
**Gender (n, %)**				0.752
Male	19 (47.5)	10 (50.0)	9 (45.0)	
Female	21 (52.5)	10 (50.0)	11 (55.0)	
**BMI** (kg/m^2^, SD)	28.4 (8.6)	25.9 (8.0)	30.9 (8.5)	0.060
**APACHE Score (SD)**	20.5 (7.6)	21.6 (8.9)	19.3 (6.1)	0.305
**Mortality Risk** (predicted)	25.0 (15.0)	32.5 (33.8)	25.0 (15.0)	0.531
**COPD (n, %)**				0.327
No	15 (37.5)	6 (30.0)	9 (45.0)	
Yes	25 (62.5)	14 (70.0)	11 (55.0)	
**CHF (n, %)**				0.749
No	23 (57.5)	12 (60.0)	11 (55.0)	
Yes	17 (42.5)	8 (40.0)	9 (45.0)	
**DM (n, %)**				1.000
No	22 (55.0)	11 (55.0)	11 (55.0)	
Yes	18 (45.0)	9 (45.0)	9 (45.0)	

BMI = Body mass index, CHF = Chronic Heart Failure, COPD = Chronic Obstructive Pulmonary Disease, DM = Diabetes Mellitus. Standard deviation (SD) and percentage are presented within brackets for continuous and categorical variables, respectively.

**Table 2:** ABGs upon admission to the Emergency Department and 24 h after hospitalization.

	Total	Patients		*p* Value
		Intervention Group	Control Group	
PaCO_2_IN (mmHg)	61.2 (10.0)	60.4 (9.9)	62.1 (10.3)	0.586
PaCO_2_ 24 h (mmHg)	54.2 (9.9)	51.6 (9.6)	56.8 (9.7)	0.096
pHIN	7.4 (0.1)	7.4 (0.1)	7.4 (0.1)	0.176
pH24 h	7.4 (0.1)	7.4 (0.1)	7.4 (0.1)	0.208
SaO_2_IN	92.3 (4.9)	92.4 (5.4)	92.1 (4.6)	0.851
SaO_2_ 24 h	93.1 (3.0)	93.3 (2.1)	92.9 (3.8)	0.644
PaO_2_IN (mmHg)	76.4 (28.9)	65.2 (12.9)	71.6 (19.8)	0.192
PaO_2_ 24 h (mmHg)	69.9 (9.7)	67.9 (8.8)	72.0 (10.4)	0.180
HCO_3_- IN (mmol/L)	36.4 (7.5)	36.7 (5.2)	36.2 (9.4)	0.836
HCO_3_- 24 h (mmol/L)	36.2 (7.8)	35.6 (7.2)	36.8 (8.5)	0.648

ABGs: Arterial Blood Gases.

**Table 3:** Length of hospitalization, complications, and mortality rate.

	Total	Patients		*p* Value
		Intervention Group	Control Group	
Length of hospitalization (days)	11.5 (8.5)	11.5 (7.8)	11.0 (10.5)	0.655
Length of 24 h uninterrupted use of HFNC or NIV (days)	2.0 (2.0)	2.0 (1.0)	2.0 (9.0)	0.078
Renal Failure				0.197
No	24 (60.0)	14 (70.0)	10 (50.0)	
Yes	16 (40.0)	6 (30.0)	10 (50.0)	
Hepatic Failure				0.698
No	36 (90.0)	18 (90.0)	18 (90.0)	
Yes	4 (10.0)	2 (10.0)	2 (10.0)	
Mortality Rate (number of deaths)				0.669
No	34 (85.0)	17 (85.0)	17 (85.0)	
Yes	6 (15.0)	3 (15.0)	3 (15.0)	

**Table 4:** Qualitative questionnaire about the use of high-flow oxygen through nasal cannula (HFNC).

Question	Scale of Available Answers	Score
1. How comfortable do you feel with the use of HFNC?	0–5 (0 = least comfortable, 5 = most comfortable)	3.9
2. How comfortable do you feel with the use of maximum available temperature (37 °C)?	0–5 (0 = least comfortable, 5 = most comfortable)	3.3
3. Which temperature is ideal for you for use with the HFNC?	31–37 °C	34.5 °C
4. How adequate is the administered air flow?	0–5 (0 = least adequate, 5 = most adequate)	4.3
5. How would you rate this type of oxygen therapy in comparison to previous oxygen therapies?	0–5 (0 = worst, 5 = best)	3
6. Rate your current degree of dyspnea.	0–5 (0 = least dyspnea, 5 = most dyspnea)	2.8
7. To what degree would you prefer HFNC compared to previous types of oxygen therapy?	0–5 (0 = I would not prefer it at all, 5 = I would prefer it absolutely)	3.1
8. Would you replace your household oxygen therapy with HFNC?	0–5 (0 = I would not replace it at all, 5 = I would replace it absolutely)	3
